# Long-Term Hematopoietic Engraftment of Congenic Amniotic Fluid Stem Cells After in Utero Intraperitoneal Transplantation to Immune Competent Mice

**DOI:** 10.1089/scd.2017.0116

**Published:** 2018-04-15

**Authors:** Panicos Shangaris, Stavros P. Loukogeorgakis, Michael P. Blundell, Eleni Petra, Steven W. Shaw, Durrgah L. Ramachandra, Panagiotis Maghsoudlou, Luca Urbani, Adrian J. Thrasher, Paolo De Coppi, Anna L. David

**Affiliations:** ^1^ Prenatal Cell and Gene Therapy Group, Institute for Women's Health, University College London, London, United Kingdom.; ^2^ Stem Cells and Regenerative Medicine, Institute of Child Health, University College London, London, United Kingdom.; ^3^ Department of Obstetrics and Gynecology, Taipei Chang Gung Memorial Hospital, College of Medicine, Chang Gung University, Taipei, Taiwan.; ^4^ Molecular and Cellular Immunology Section, Institute of Child Health, University College London, London, United Kingdom.; ^5^ NIHR University College London Hospitals Biomedical Research Centre, London United Kingdom.

**Keywords:** amniotic fluid stem cells, prenatal therapy, in utero stem cell transplantation

## Abstract

Clinical success of in utero transplantation (IUT) using allogeneic hematopoietic stem cells (HSCs) has been limited to fetuses that lack an immune response to allogeneic cells due to severe immunological defects, and where transplanted genetically normal cells have a proliferative or survival advantage. Amniotic fluid (AF) is an autologous source of stem cells with hematopoietic potential that could be used to treat congenital blood disorders. We compared the ability of congenic and allogeneic mouse AF stem cells (AFSC) to engraft the hematopoietic system of time-mated C57BL/6J mice (E13.5). At 4 and 16 weeks of age, multilineage donor engraftment was higher in congenic versus allogeneic animals. In vitro mixed lymphocyte reaction confirmed an immune response in the allogeneic group with higher CD4 and CD8 cell counts and increased proliferation of stimulated lymphocytes. IUT with congenic cells resulted in 100% of donor animals having chimerism of around 8% and successful hematopoietic long-term engraftment in immune-competent mice when compared with IUT with allogeneic cells. AFSCs may be useful for autologous cell/gene therapy approaches in fetuses diagnosed with congenital hematopoietic disorders.

## Introduction

In humans, successes with in utero transplantation (IUT) using allogeneic hematopoietic stem cells (HSCs) has been limited to fetuses with severe immunologic defects, where there is an effective lack of immune response to the transplanted allogeneic cells, and where these genetically normal cells have a proliferative advantage [1]. Mesenchymal stem cells (MSCs) appear to be less immunogenic than their hematopoietic counterparts. They engraft after IUT in human fetuses with osteogenesis imperfecta in an allogeneic setting [2] and reduce fracture rate in a mouse model of the disease [3]. However, attempts to treat congenital hematological diseases such as sickle cell disease [4] with in utero HSC transplantation have been unsuccessful, even where a suitably matched donor has been available.

Studies in mice suggest that the immune barrier to allogeneic in utero HSC transplantation may be stronger than previously thought [5,6]. Maternal antibodies also play an important role [7,8]. Even though induction of tolerance for allogenic cells could be a promising approach [9], transplantation of autologous progenitor cells, which have been corrected for congenital disease, could avoid the fetal immune barrier.

Amniotic fluid (AF) is a source of ckit^+^ stem cell-like progenitors with hematopoietic potential that are characterized by long-term self-renewal and the ability to differentiate into all three lineages [10–13]. AF can be collected using a clinically safe ultrasound guided method, currently used for prenatal diagnosis of congenital disease. AF stem cells (AFSCs) from mice and humans can be expanded as MSCs without feeder layers [14], are not tumorigenic, and retain long telomeres and a normal karyotype for over 250 population doublings [15,16] Their potential for gene transfer [17] prenatal and postnatal therapy is enormous [18,19]. We have previously demonstrated the potential of AFSCs for autologous IUT in pregnant sheep, a clinically relevant large animal model whereby mesenchymal or hematopoietic selected AFSCs engrafted after autologous transplantation in utero [20,21]. We hypothesize that AF-derived stem cells could be used as an autologous source of cells for the treatment of congenital hematological disease [19].

Here, we studied the engraftment and immune response to cKit^+^Lin^−^ AF cells after congenic and allogeneic IUT in mice using intraperitoneal injection, a clinically relevant delivery method that has been safely used in the first trimester for IUT in humans [1].

## Materials and Methods

### Animal care

All the animals used were sourced from Harlan Company and were housed in a single cage after plugging. Vaginal plugs were checked by the animal technicians and the first day of gestation was considered the day after the plug was found. The dams were given wet food after surgery and closely monitored for the first 7 days after the procedure. The animal experiments were done according to the project license PPL No: 70/7408 and Animals [Scientific Procedures] Act 1986 and the NC3Rs ARRIVE guidelines 2013.

### Cell sources

Mouse AFSCs (mAFSC) were isolated by lineage depletion with magnetic separation (Miltenyi Biotec), followed by CD117 separation using flow cytometry as described [13]. Similarly bone marrow (BM) stem cells were isolated from mouse adult BM using published techniques for lineage depletion with magnetic separation (Miltenyi Biotec) followed by separation using flow cytometry and Sca1 antibody. Lin^−^/Sca1^+^ cells were used for BM transplantation [8,22]. Fetal liver (FL) stem cells were used for comparison. These were isolated from E13.5 fetuses by mechanical dissociation of the FL and lineage depletion by magnetic separation. The cells were characterized using flow cytometry to demonstrate the specific cell populations used for the control experiments and IUT (Supplementary Fig. S1; Supplementary Data are available online at www.liebertpub.com/scd). To mimic autologous IUT, cKit^+^/lin^−^, congenic mAFSC, were isolated at E13.5 from B6.SJL-Ptprca Pepcb/BoyJ dams (CD45.1; MHC class I: H2-K^b+^). For allogeneic IUT, mAFSC were isolated from fetuses of BALB/cJ (CD45.2^+^; MHC class I: H2-K^d+^) dams. After confirming death, the abdomen of the mouse was cleaned and carefully opened with sterile scissors and the uterine horns were removed intact into a sterile petri dish. The uterus was irrigated with phosphate-buffered saline (PBS) to remove maternal blood. Using sterile scissors, each amniotic sac was carefully dissected from the surrounding uterus and washed with PBS. The amniotic sac was then punctured using fine sterile scissors and the whole AF (about 50 μL) was collected into a 20 mL falcon tube after passing through a 50 μL nylon filter. Only clear AF was collected. Obviously blood stained AF was discarded to exclude maternal or placental contamination as described [13].

The clear AF from all fetuses from up to three dams was pooled together. Red Blood Cell lysis was used to remove any remaining red blood cells and to prevent any fetal or maternal blood contamination. The cells were then washed with PBS three times and counted. IUT was performed at E13.5 into C57BL/6 (CD45.2/H-2K^D^) dams time mated with C57BL/6 (CD45.2/H-2K^D^) male mice. There were at least three different dams transplanted in three biologically independent experiments in each group.

### In utero stem cell transplantation

Mouse IUT as performed at E13.5 under general anesthesia. After clipping the abdominal wall, cleaning, and sterile draping, the abdomen was opened by a midline incision to reveal the two pregnant uterine horns. At laparotomy, the liver of each embryo was identified through the transparent uterine wall. Prepared mAFSCs or Congenic BM Stem Cells in 20 μL (1 × 10^4^ or 5 × 10^4^ cells) of PBS were injected into the peritoneal cavity inferior to the liver of each embryo using a 33-gauge needle attached to a digital syringe (Hamilton, Switzerland). All embryos of each litter were injected. The abdomen was closed in two layers using 6.0 Vicryl Rapide suture (Ethicon). The skin layer was closed using the subcuticular method. The dams were transferred to a warm cage (28°C) for initial recovery, given wet food and closely monitored postoperatively.

After spontaneous delivery at E20–E21 day of gestation, treated pups were cross-fostered to CD1 after birth, to avoid maternal cannibalism and maternal antibody response to the transplanted cells [8,23,24]. They underwent a scheduled postmortem examination at 4 or 16 weeks after birth, where blood and tissue were isolated for flow cytometry, in vitro mixed lymphocyte reaction (MLR) histology, and qPCR as described below.

### Blood collection

Blood was collected under terminal anesthesia, induced by injecting tribromoethanol (0.8 mL of 1.25%; Avertin Sigma- Aldrich) solution into the peritoneal cavity. About 0.5–1 mL of blood was collected into 0.5 mL EDTA tubes by cardiac puncture using a preheparinized 26G syringe.

### Tissue isolation

At scheduled postmortem examination, the animals were culled and the thoracic and abdominal cavity exposed by a midline incision. The organs (spleen, kidneys, heart, lungs, and liver) were removed using sterile forceps and scissors and placed in microtubes (1.5 mL Safe-Lock; Eppendorf) for RNA isolation. Small amounts of tissue were collected into sterile PBS for flow cytometry analysis. Also, tissue sections were fixed in 4%PFA for histological analysis. For BM collection, the muscle tissue was cleared from the femoral and long tibia bones; the epiphyses were removed and placed in microtubes (1.5 mL Safe-Lock; Eppendorf).

### Blood and tissue staining for flow cytometry

The tissues (liver and spleen) were initially mechanically dissociated using a 40 mm nylon filter. The BM was flushed out of the long bones during PBS and 1 mL syringe with 26G needle. The sampled blood or dissociated tissues were added to 5 mL RBC Lysis Buffer in conical tubes (15 mL, Falcon; BD Biosciences). These were then centrifuged at 300 *g* for 5 min. The lysate was aspirated and resuspended in 100 μL Flow Cytometry PBS, PH7.2, with 0.5% of Bovine Serum Albumin (BSA) (SB buffer). One μL of the conjugated antibody was then added and incubated at 4°C for 15 min. After 15 min the lysate was washed with 1–2 mL of SB buffer and spun for 5 min at 300 *g*. The supernatant was discarded. The pellet was transferred to a flow cytometry tube (5 mL; BD Biosciences) after resuspension with 500 μL of SB Buffer and then analyzed using the flow cytometry analyzer LSR II (BS Biosciences).

For the detection of the transplanted cells a specific antibody against the donor cells was used as follows: for congenic experiments, CD45.1 (Fig. 2A, C, E) and for allogenic experiments H-2K^d^ (Fig. 2B, D, F). The results are presented as the number of positive cells for the donor antibody out of the total number of CD45^+^ cells (Supplementary Fig. S2 for the gating strategy used). Animals injected with PBS were used as flow cytometry controls. In the erythroid differentiation assay, mouse embryonic fibroblasts were used as negative controls. For the lineage analysis, the lineage-specific antibodies CD3, CD11b, B220, Gr1, and Ter-119 (Miltenyi Biotec, Germany) were used. The hematopoietic colonies were liquefied using RPMI 1640 (Thermo Fisher Scientific) and stained with donor markers before flow cytometry. The viability dye 7-Amino-Actinomycin D (7AAD) (Sigma-Aldrich) was used to exclude dead cells from the analysis.

### In vitro MLR

The in vitro MLR assay was performed as published [25], in three different animals of each group in triplicates. For the proliferation assays, splenocytes from recipients of congenic and allogenic transplants were labeled with the dye carboxyfluorescein diacetate succinimidyl ester (CFSE; Invitrogen) by incubating cells in CFSE (1 μM; Invitrogen) in 1 mL PBS at 37°C for 10 min, followed by three washes in RPMI with 10% FBS.

One milliliter of medium containing labeled cells were added to 96-well U-bottom plates at a concentration of 1,000,000 cells/mL in RPMI culture media [10% FBS, 2 mM L-Glu, and 100 U/mL penicillin/streptomycin (Invitrogen Life Sciences)]. For both groups, allogeneic and congenic MLRs, cells from uninjected (naïve) BALBc and CD45.1 animals, unlabeled splenocytes/lymphocytes, were irradiated (6,000 rads from a cesium source, = 60 Gy × 33 s = 1,980 s) and added to the wells for a final ratio of 1:1.

The CFSE-labeled lymphocytes from the transplant recipients were incubated for 5 days (120 h) with the unlabeled irradiated naïve splenocytes/lymphocytes The cells were washed and prepared for flow cytometry analysis by incubation with the antibodies CD3, CD4, and CD8 (Miltenyi Biotec). The viability dye 7-Amino-Actinomycin D (7AAD) (Sigma-Aldrich) was used to exclude dead cells from the analysis.

### Erythroid differentiation

The erythroid differentiation was performed by culturing the cells in a concentration of 100,000 cells per 1.9 cm^2^, 52,631 cells/cm^2^ in erythroid differentiation medium containing 50 mL stem span (Stem Cell Technologies) 10 mL FBS, dexamethasone (Sigma-Aldrich), 1 mL of 100%EtOH in 50 mL of medium (20 mcg/mL medium concentration), 10 μL of erythropoietin (EPO, 10 μL/mL; Sigma-Aldrich), and 100 μL of stem cell factor (SCF, 1 mg/mL; Sigma-Aldrich). The cells were plated in the erythroid medium for a total of 5 days before flow cytometry analysis was conducted for the presence of adult hemoglobin using a specific adult hemoglobin antibody (Hb-PE; Abcam).

### Semi-solid colony forming assays

The semi-solid assays were performed as per manufacturer's instructions by culturing 10^4^–10^5^ cells expressing the donor's marker (CD45.1^+^ or H2-K^d+^) per 35 mm dish. The cells were isolated from the BM of the transplant recipients or freshly isolated AFSCs at E13.5. The cells were prepared, washed and counted, and were added to a 5 mL tube containing 2.5 mL of MethoCult (MethoCult™ GF M3434, Stem Cell Technologies, containing Methylcellulose in Iscove's MDM, Fetal bovine serum, Bovine serum albumin, Recombinant human insulin, Human transferrin (iron-saturated), 2-Mercaptoethanol, Recombinant mouse SCF, Recombinant mouse interleukin 3 (IL-3), Recombinant human interleukin 6 (IL-6), and Recombinant human EPO. The tube was vortexed and allowed to stand to allow bubbles to dissipate. The cells were then dispensed into 35 mm culture dishes using a syringe and a blunt needle. The cells were incubated for 14 days in a humidified incubator at 37°C and 5% CO_2_. The colonies were then counted using an inverted microscope and gridded scoring dishes. The culture was liquefied using RPMI medium (GIBCO) for further flow cytometry processing.

### Real-time PCR

The expression levels of FoxP3, TGF- β, and IL-10 was analyzed using the SensiFAST SYBR Hi-ROX One-Step Kit (Bioline) as per manufacturer's instructions amplifying 10 ng template RNA. The relative expression levels in wild-type C57BL/6J mice of Foxp3 and TGF-β were used for normalization of results.

Relative quantification of each target gene studied was calculated by the ΔΔCt method on an Applied Biosystem Step One Plus Real-Time PCR system (ThermoFisher Scientific). The forward and reverse primers used can be found in Supplementary Table S1 and they were adapted from Singh et al. [26].

### Statistical analysis

Statistical analysis used GraphPad Prism 7 with ANOVA as the main statistical test including Bonferroni correction. The numbers are presented as mean ± SEM. To achieve a statistically meaningful result, for each group we aimed to have at least six mice pups in each group, born by more than three different dams, to have more than three independent biological replicates.

## Results

### Mouse AFSC can produce hematopoietic colonies and hemoglobin in vitro

**FIG. 1.**
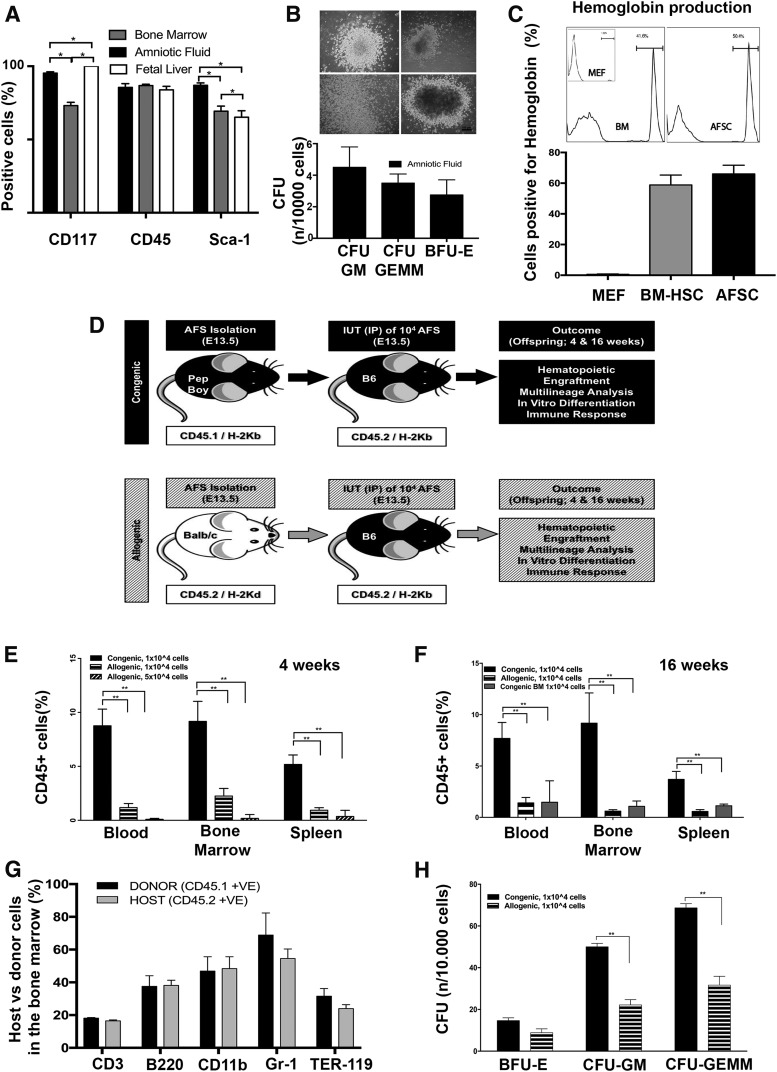
Amniotic fluid stem cell isolation, in utero stem cell transplantation and in vitro differentiation. **(A)** Flow cytometry expression analysis (purity) of CD117, CD45 and Sca1 in the freshly isolated cells FL: CD117 (100 ± 0.0), CD45 (83.85 ± 2.45), Sca1(65.20 ± 4.4) (BM: CD117 (73.10 ± 2.30), CD45 (86.80 ± 0.90), Sca1(69.30 ± 3.5), AFSC: CD117 (95.25 ± 0.95), CD45 (85.5 ± 2.55), Sca1(86.95 ± 1.65) There was no significant difference in CD45 expression, *n* = 6, *p* = 61. **(B)** Mouse AFSCs successfully produced hematopoietic colonies (CFU-GM, CFU-GEMM, BFU-E, *n* = 3) after 2 weeks in semisolid medium (Methocult, M3434, stem cell technologies, Scale Bar 100 μm). **(C)** Hemoglobin production of AFSC after culture in a terminal erythroid differentiation medium for 5 days. BM and mouse embryonic fibroblasts were used as a control (*n* = 3). **(D)** Study design all embryos in each litter were successfully injected, and all dams survived the surgical procedure. **(E)** Engraftment (measured as percentage of donor cells in total of CD45^+^ cells) at 4 weeks after birth in the congenic versus the allogeneic group in the blood (8.191 ± 1.45, *n* = 7 vs. 1.18 ± 0.37, *n* = 8, *P* < 0.05), bone marrow (8.05 ± 1.96, *n* = 7 vs. 2.25 ± 0.70, *n* = 8, *P* < 0.05), and spleen (4.67 ± 0.92, *n* = 7 vs. 0.94 ± 0.23, *n* = 8, *P* < 0.05). Transplanting a fivefold higher number of cells (5 × 10^4^) in recipients of the allogenic group did not increase engraftment blood 0.07 ± 0.02, bone marrow 0.13 ± 0.11, and spleen 0.28 ± 0.18, *n* = 6. **(F)** Engraftment (measured as percentage of donor cells in total of CD45^+^ cells) at 16 weeks in the blood (congenic AFSC 7.69 ± 1.55, *n* = 11 vs. allogenic ASFC, *n* = 9 1.41 ± 0.52 vs. congenic bone marrow *n* = 4 1.47 ± 1.20, *P* < 0.05), bone marrow (9.17 ± 2.94 vs. 0.60 ± 1.41 vs. 1.07 ± 0.26, *P* < 0.05), and spleen (3.69 ± 0.78 vs. 0.58 ± 0.17 vs. 1.13 ± 0.09, *P* < 0.05). **(G)** Donor cells (CD45.1 and H2K^d^) from the bone marrow at 16 weeks were positive for all three blood lineages (% of lineage marker within CD45^+^ cells) (CD3, B220, CD11b, Gr1, and Ter119) and not significantly different from the host cell populations (CD45.2 and H2K^b^) (*n* = 3, *P* = 0.99). **(H)** After culturing the donor cells in a semisolid differentiation medium, there was a significantly higher number of colonies in congenic versus allogenic cell sources (BFU-E 14.67 vs. 8.83 *P* = 0.331, CFU-GM 50.00 vs. 22.17, *P* < 0.05, CFU-GEMM 68.67 vs. 31.67, *n* = 3, *P* < 0.05). *P*-values *, and ** denote levels 0.05 and 0.01 of statistical significance accordingly. AFSC, amniotic fluid stem cell; BM, bone marrow.

We observed that fresh AFSCs can be reliably isolated from mouse AF and have equal CD45 expression to mouse FL-derived HSC and BM HSC while differing from FL-HSC for Sca-1 expression and from BM-HSC for cKit and Sca-1 (Fig. 1A and Supplementary Fig. S2 for co-expression of stem cell markers). AFSC cell cycle analysis demonstrated as expected that the clear majority of cells were in GO/G1 phase (78.53% ± 3.85%), with the rest of the cells distributed in S (9.85% ± 2.21%) and G2 M phase (6.51% ± 0.80%) *n* = 3. When cultured in semi-solid medium mAFSCs produced hematopoietic colonies. (Fig. 1B). Further differentiation pushed them to terminal erythrocytes with mouse hemoglobin production at levels comparable to an adult mouse (Fig. 1C).

### Congenic mouse AFSCs engraft in the immune competent mouse in utero

Having demonstrated their hematopoietic potential in vitro, mAFSCs were transplanted in utero to immunocompetent mice via intraperitoneal injection, to test their ability to engraft long term (Fig. 1D). All embryos of each litter were injected. After birth, pups were cross-fostered in the day after birth. Pup survival rate was comparable between the two groups (18 pups out of 37, 48.6%: congenic, 17 out of 36, 47.2%: allogeneic). Immediate neonatal death was due to maternal cannibalism seen when pups were cross-fostered, or due to in utero fetal demise post IUT, which occurred in approximately 30% of injected pups. (evidence of miscarriage was seen during the postmortem analysis of the dam that was performed after cross-fostering). There were no pup deaths from cross-fostering up to scheduled postmortem examination at 4 or 16 weeks.

All congenic transplanted animals (18 out of 18) were chimeric (>1% of positive donor cells among the CD45^+^ cells population) compared with only 29% (5 out of 17) of the animals receiving allogeneic cells. Compared to allogeneic transplantation of AFSCs (Ckit^+^/Lin^−^) and Congenic BM (Sca1^+^/Lin^−^), autologous transplantation of AFSCs resulted in far higher levels of engraftment in animals analyzed at 4 or 16 weeks, in the blood, BM, and spleen (Fig. 1E, F). Increasing the number of injected cells by fivefold (5 × 10^4^) did not improve engraftment of allogeneic AFSCs; indeed, engraftment was even lower (Fig. 1E).

### Mouse AFSCs show hematopoietic multilineage engraftment

Donor-recipient balanced multilineage long-term engraftment was confirmed in the BM of recipient groups at 16 weeks postnatal by characterization of donor cell marker expression (CD45.1 and H2K^d^) using donor-specific antibody with hematopoietic lineage markers CD3, B220, CD11b, Gr1, and Ter-119 i.e., cells double positive for the donor specific and lineage marker (Fig. 1G).

Cells positive for donor markers from 16-week postnatal recipient BM were selected using FACS and cultured in a semi-solid medium for 2 weeks. The culture confirmed the presence of hematopoietic colonies from blood lineages: erythroid & myeloid (Fig. 1H). There were a higher number of CFU-GM (Colony Forming Unit, Granulocyte–Monocyte) and CFU-GEMM (Colony Forming Unit, Granulocyte, Erythrocyte, Monocyte, Megakaryocyte) colonies generated from BM of animals injected with congenic AFSCs when compared with BM of animals injected with allogeneic AFSCs. The colonies were liquefied using flow RPMI medium and flow cytometry analysis confirmed presence of donor cells (CD45.1^+^ and H2K^d+^). The liquefied colonies were also analyzed using the differentiation markers CD3, B220, CD11b, Gr1, and Ter-119 (Supplementary Fig. S3). The results show the presence of donor cells and markers of terminal hematopoietic differentiation in the liquefied colonies.

### Absent immune response to congenic but not allogenic mouse AFSCs in transplant recipients

**FIG. 2.**
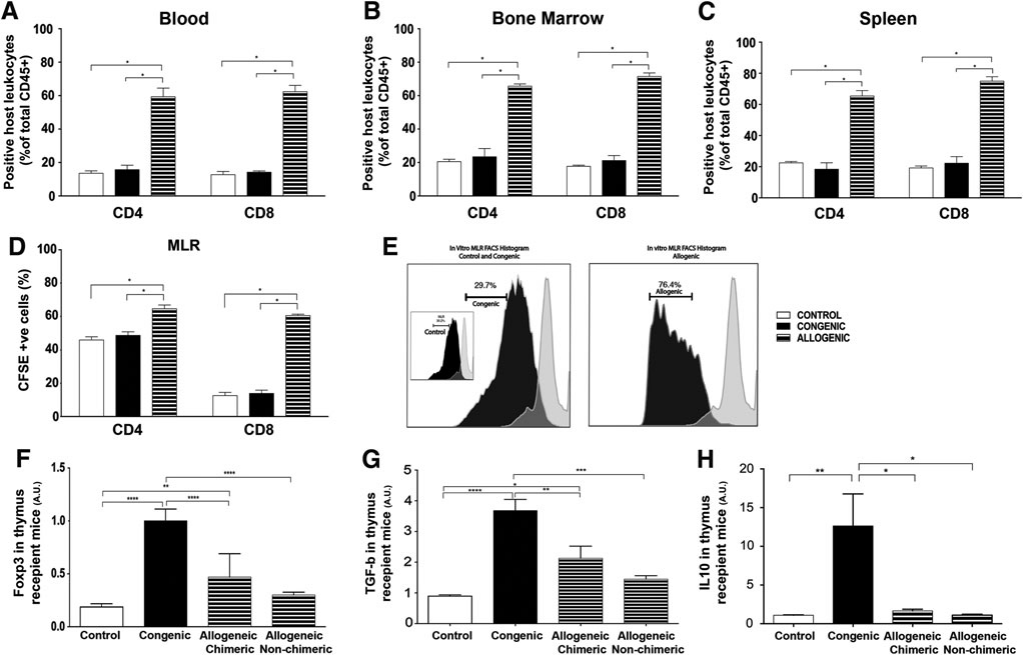
Immune response to allogenic stem cell transplantation. **(A–C)** Compared with control and congenic cell transplanted groups, there was a significantly higher percentage of CD4 and CD8 cells per total CD45^+^ count in the allogenic transplanted group, in the blood (CD4:13.57 ± 1.44 vs. 15.70 ± 2.67 vs. 59.33 ± 5.15, CD8: 12.70 ± 1.94 vs. 14.20 ± 0.73 vs. 62.37 ± 3.77), bone marrow (CD4:20.50 ± 1.42 vs. 23.43 ± 4.94 vs. 65.67 ± 1.33, CD8:17.70 ± 0.73 vs. 21.16 ± 2.94 vs. 71.50 ± 2.09), and spleen (CD4: 22.43 ± 0.95 vs. 18.36 ± 4.16 vs. 65.40 ± 3.50, CD8: 19.17 ± 1.29 vs. 22.23 ± 4.23 vs. 74.96 ± 2.83) There was no significant difference between the congenic and control transplanted groups (*n* = 3, *P* = 0.99). **(D, E)** T cell proliferation of recipient CSFE labeled splenocytes stimulated with inactivated splenocytes from the donor was significantly higher in the allogenic group (CD4 = 64.53% ± 2.28%, CD8 = 60.48% ± 0.82%, *P* < 0.05) with no difference seen after stimulation in the control transplanted (CD4 = 46.07% ± 1.61%, CD8 = 12.59% ± 1.93%, *P* > 0.99) and the congenic transplanted group (CD4 = 48.57 ± 2.11, CD8 = 13.93% ± 1.94%, *P* > 0.99). **(F)** Relative gene expression of Foxp3 by qRT-PCR in the thymus was significantly higher in the congenic compared to the allogenic chimeric animals at 4 weeks. Congenic versus allogenic chimeric (1.0 vs. 0.47, *n* = 8, *P* < 0.05), congenic vs allogenic nonchimeric (1.0 vs. 0.30, *n* = 4, *P* < 0.05), congenic versus control (1.0 vs. 0.19, *n* = 7, *P* < 0.0001) and Allogenic chimeric versus control animals (0.47 vs. 0.19, *n* = 8, *P* < 0.05). **(G)** Similar to Foxp3, relative gene expression of TGF-beta by qRT-PCR in the thymus was significantly higher in the congenic compared to the allogenic chimeric animals. Differences were seen in the congenic versus control (0.90 vs. 3.7, *n* = 7, *P* < 0.05), allogenic chimeric versus control (2.1 vs. 0.90, *n* = 8, *P* < 0.05), congenic versus allogenic chimeric (3.7 vs. 2.1, *n* = 8, *P* = 0.0025) and congenic versus allogenic nonchimeric (3.7 vs. 1.4, *n* = 4, *P* < 0.05). **(H)** There was higher IL10 gene expression in the congenic group compared to other groups and the control (12.64 vs. 1.095 vs. 1.66 vs. 1.10, *n* = 5, *P* < 0.05). *P*-values *^,^ **^,^ *** and **** denote levels 0.05, 0.01, 0.001 and 0.0001 of statistical significance accordingly.

To further understand the reasons for the low engraftment seen in the allogeneic recipient group we investigated the role of the immune response to IUT. There was no difference in the total number of CD4 and CD8 cells present at 4 weeks in control and autologous recipient BM, blood, and spleen, but the total CD4 and CD8 cell numbers were far higher in the allogenic recipient group (Fig. 2A–C). Similarly, in vitro MLR studies showed a significantly higher rate of CD4 and CD8 T lymphocyte proliferation in the splenocytes of the allogeneic recipient group compared with the control and congenic recipient group Fig. 2D and E.

In the spleen, in the congenic recipient animals the relative expression of Foxp3 was significantly increased (*P* < 0.05) (4.94 ± 1.12, *n* = 4) while the allogenic recipient animals had a lower mean expression (0.05 ± 0.015, *n* = 5). In the congenic recipient animals, the relative expression of Foxp3 in the BM was significantly increased (*P* < 0.05) (1.71 ± 0.25, *n* = 4) compared with allogenic recipient animals (0.035 ± 0.0086, *n* = 5).

In the spleen, in the congenic group (3.17 ± 0.53, *n* = 4) the relative expression of TGF- β was significantly increased (*P* < 0.05) versus allogenic group (0.014 ± 0.0065, *n* = 5). In the congenic group the relative expression of TGF-β in the BM was significantly increased (9.46 ± 0.84, *n* = 4) versus allogenic animals (0.76 ± 0.28, *n* = 5).

The relative gene expression of FoxP3, TGF-β, and IL-10 at 4 weeks was higher in the thymus, spleen, and BM of congenic compared with allogeneic recipients, suggesting a higher acceptance of the graft and lack of an immune response. There was no significant difference in the gene expression between allogeneic chimeric and allogenic nonchimeric recipients (Data shown only for thymus; Fig. 2F–H).

### Histological analyses

Tissues from the IUT recipient animals showed no evidence of pathological changes.

## Discussion

We investigated the potential barriers to the in utero engraftment of AFSCs in this study. We first confirmed that stem cells can be isolated from mouse AF as previously described [13,15] and found that most of the purified AFSCs are in the G0/G1 phase of the cell cycle corresponding to a primitive HSC with high stem cell activity [27], which are in cell quiescence.

To ensure as much as possible that the AF was free from contamination with maternal cells we collected the fluid using a well-described technique [13]. First the uterus and then the amniotic sacs were carefully washed before puncture of the amniotic membrane with a sterile technique, ensuring as far as possible that only AF was isolated from the amniotic sac. Obviously, contaminated samples were rejected and the fluid was then subjected to red cell lysis to destroy any contaminating fetal or maternal red blood cells. In our previous study, GFP expression was used to select donor AF cells before transplantion [13]. In this study it was not possible to use the same technique as the experimental set up involved different mouse strains. Nevertheless, we believe that our collection technique is robust for selecting fetal cells. This is supported by the findings from our control group experiments in which maternal BM-derived stem cells were found to have minimal engraftment.

When cultured in the semi-solid medium the mouse AFSCs produced hematopoietic colonies from all three hematopoietic lineages, and they were capable of hemoglobin production when cultured in an erythroid differentiation medium similarly to BM stem cells [28]. Having demonstrated their hematopoietic potential in vitro, the mouse AFSCs were used in an in utero stem cell approach to test their ability to engraft long term. We found that allogeneic transplantation in utero was associated with lower rats of engraftment at 4 and 16 week time points, when compared with autologous transplantation. This was of interest to us, as an in utero approach is considered to be relatively more successful at achieving engraftment through fetal tolerance [6,9,29–32]. Injecting even higher numbers (5 × ) of allogenic AFSC cells did not achieve tolerance or higher engraftment. We ensured that there was no maternal antibody effect postnatally by cross-fostering the pups to different dams after birth, thus avoiding passage of antibodies through lactation. This effect has previously been described in mice and nevertheless we cannot rule out the possibility that maternal antibodies passed across the placental barrier following allogenic IUT and before birth. The lack of tolerance might also be due to the primitive nature of the ASFC and their inability to present in the thymus and achieve a “self” identity [33–35].

Due to the low engraftment seen in the allogenic transplanted group we studied the immune reaction using the total number of CD4 and CD8 cells present at 4 weeks. The presence of an immune response was confirmed by performing in vitro MLR studies where splenocytes (responders) isolated from the IUT recipient mice at 4 weeks of age were cocultured with splenocytes from genetically identical naïve donors (stimulators). The significantly higher proliferation rate of CD4 and CD8 T lymphocytes in the allogenic group confirmed the presence of an immune response toward the donor cells. On the contrary, in the congenic group the T lymphocyte proliferation was similar to the uninjected control, which indicates that the congenic cells did not form any significant negative antigen response. The congenic recipients did have a tolerogenic response though, since FoxP3, TGF-β, and IL10 gene expression were significantly higher in comparison with the uninjected control and allogenic group when measured by qPCR.

Multilineage long-term engraftment was confirmed in the recipient groups by characterization of the cells expressing the donor markers using hematopoietic lineage markers such as CD3, B220, CD11b, Gr1, and Ter-119. We detected double positive CD45.1 and lineage-specific cells in BM and blood, which is an indication that mouse AFSCs can give rise to terminally differentiated cells and integrate fully into the hematopoietic system. The cells were also cultured in a semi-solid medium, which confirmed the multilineage engraftment by the presence of hematopoietic colonies from blood lineages, myeloid, and erythroid. The hematopoietic colonies were also analyzed using flow cytometry, which confirmed expression of differentiation markers [6,31]. The higher expression of FoxP3, TGF-β, and IL-10 both centrally and peripherally in the congenic recipients suggests the possibility that the congenic cells expressing the markers CD45.1 lead to a more tolerant state toward the transplanted cells. Studies have shown that increased FoxP3 expression is associated with self-tolerance and tolerance to noninherited antigen [36]. In addition CD4^+^CD25^+^FoxP3^+^ regulatory T cells are involved in transplantation tolerance and they prevent rejection in solid organ transplantation when co-transplanted with the allograft [37,38].

For clinical translation, we have demonstrated that HSCs derived from the AF could be used as a source for the treatment of congenital blood disorders. Since allogeneic AFSCs appear to cause an immune response in mice, autologous (congenic) transplantation would be preferred. After combining gene therapy and subsequent correction and expansion of gene-corrected AFSCs, the cells could be given back to the same fetus with the aim to correct hematological or other monogenic disease before birth [17].

The multilineage engraftment we observed suggests that these cells could reconstitute the hematopoietic system if given in sufficient numbers. AFSC expansion while maintaining their hematopoietic potential is an important hurdle that will need to be overcome to provide sufficient stem cells to cure hematopoietic disease. In addition, the safety of gene transfer to AFSC for long-term transduction will need to be assessed. Transplantation of induced pluripotent stem cells from AF or other fetal sources such as chorionic villi, if proven safe, could be another approach. The next step is to assess the engraftment potential of AFSC after IUT using other injection methods such as intravenous umbilical vein injection that is also applicable to human pregnancies, and to study in detail the immune response(s) to allogeneic and congenic AFSC sources. Another limitation to the study is that we did not demonstrate whether transplanted AFSCs were able to function in vivo, for example, to produce hemoglobin, and potentially correct a mouse model of congenital disease such as thalassemia. Such experiments are being planned.

In conclusion, we have shown successful hematopoietic engraftment in immune-competent mice using mouse AFSCs. Production of hemoglobin in vitro shows that these cells are functional, forming terminal erythrocytes. Congenic AFSCs appear to have a significant engraftment advantage over allogenic AFSCs, which may be mediated by a reduced fetal immune response.

## Supplementary Material

Supplemental data

Supplemental data

Supplemental data

Supplemental data

## References

[1] Tiblad E and Westgren M (2008). Fetal stem-cell transplantation. Best Pract Res Clin Obstet Gynaecol 22:189–201 18035592 10.1016/j.bpobgyn.2007.07.007

[2] Götherström C, Westgren M, Shaw SWS, Aström E, Biswas A, Byers PH, Mattar CNZ, Graham GE, Taslimi J, et al. (2014). Pre- and postnatal transplantation of fetal mesenchymal stem cells in osteogenesis imperfecta: a two-center experience. Stem Cells Transl Med 3:255–264 24342908 10.5966/sctm.2013-0090 PMC3925052

[3] Guillot PV, Abass O, Bassett JHD, Shefelbine SJ, Bou-Gharios G, Chan J, Kurata H, Williams GR, Polak J and Fisk NM (2008). Intrauterine transplantation of human fetal mesenchymal stem cells from first-trimester blood repairs bone and reduces fractures in osteogenesis imperfecta mice. Blood 111:1717–1725 17967940 10.1182/blood-2007-08-105809

[4] Westgren M, Ringden O, Eik-Nes S, Ek S, Anvret M, Brubakk AM, Bui TH, Giambona A, Kiserud T, et al. (1996). Lack of evidence of permanent engraftment after in utero fetal stem cell transplantation in congenital hemoglobinopathies. Transplantation 61:1176–1179 8610414 10.1097/00007890-199604270-00010

[5] Peranteau WH, Endo M, Adibe OO, Merchant A, Zoltick PW and Flake AW (2006). CD26 inhibition enhances allogeneic donor-cell homing and engraftment after in utero hematopoietic-cell transplantation. Blood 108:4268–4274 16954501 10.1182/blood-2006-04-018986 PMC1895454

[6] Peranteau WH, Endo M, Adibe OO and Flake AW (2006). Evidence for an immune barrier after in utero hematopoietic-cell transplantation. Blood 109:1331–1333 17023584 10.1182/blood-2006-04-018606 PMC1785153

[7] Nijagal A, Wegorzewska M, Le T, Tang Q and MacKenzie TC (2011). The maternal immune response inhibits the success of in utero hematopoietic cell transplantation. Chimerism 2:55–57 21912720 10.4161/chim.2.2.16287 PMC3166485

[8] Merianos DJ, Tiblad E, Santore MT, Todorow CA, Laje P, Endo M, Zoltick PW and Flake AW (2009). Maternal alloantibodies induce a postnatal immune response that limits engraftment following in utero hematopoietic cell transplantation in mice. J Clin Invest 121:582–592 10.1172/JCI38979 PMC2735937 19652363

[9] Peranteau WH, Hayashi S, Abdulmalik O, Chen Q, Merchant A, Asakura T and Flake AW (2015). Correction of murine hemoglobinopathies by prenatal tolerance induction and postnatal nonmyeloablative allogeneic BM transplants. Blood 126:1245–1254 26124498 10.1182/blood-2015-03-636803 PMC4559936

[10] Prusa A-R, Marton E, Rosner M, Bernaschek G and Hengstschläger M (2003). Oct-4-expressing cells in human amniotic fluid: a new source for stem cell research? Hum Reprod 18:1489–1493 12832377 10.1093/humrep/deg279

[11] Fauza D (2004). Amniotic fluid and placental stem cells. Best Pract Res Clin Obstet Gynaecol 18:877–891 15582544 10.1016/j.bpobgyn.2004.07.001

[12] De Coppi P, Callegari A, Chiavegato A, Gasparotto L, Piccoli M, Taiani J, Pozzobon M, Boldrin L, Okabe M, et al. (2007). Amniotic fluid and bone marrow derived mesenchymal stem cells can be converted to smooth muscle cells in the cryo-injured rat bladder and prevent compensatory hypertrophy of surviving smooth muscle cells. J Urol 177:369–376 17162093 10.1016/j.juro.2006.09.103

[13] Ditadi A, de Coppi P, Picone O, Gautreau L, Smati R, Six E, Bonhomme D, Ezine S, Frydman R, Cavazzana-Calvo M and André-Schmutz I (2009). Human and murine amniotic fluid c-Kit+Lin- cells display hematopoietic activity. Blood 113:3953–3960 19221036 10.1182/blood-2008-10-182105

[14] Piccoli M, Franzin C, Bertin E, Urbani L, Blaauw B, Repele A, Taschin E, Cenedese A, Zanon GGF, et al. (2012). Amniotic fluid stem cells restore the muscle cell niche in a HSA-Cre, SmnF7/F7 mouse model. Stem Cells 30:1675–1684 22644669 10.1002/stem.1134

[15] De Coppi P, Bartsch G, Siddiqui MM, Xu T, Santos CC, Perin L, Mostoslavsky G, Serre AC, Snyder EY, et al. (2007). Isolation of amniotic stem cell lines with potential for therapy. Nat Biotechnol 25:100–106 17206138 10.1038/nbt1274

[16] Loukogeorgakis SP and De Coppi P (2016). Stem cells from amniotic fluid - Potential for regenerative medicine. Best Pract Res Clin Obstet Gynaecol 31:45–57 26542929 10.1016/j.bpobgyn.2015.08.009

[17] Grisafi D, Piccoli M, Pozzobon M, Ditadi A, Zaramella P, Chiandetti L, Zanon GF, Atala A, Zacchello F, et al. (2008). High transduction efficiency of human amniotic fluid stem cells mediated by adenovirus vectors. Stem Cells Dev 17:953–962 18564037 10.1089/scd.2007.0188

[18] Shaw SWS, David AL and De Coppi P (2011). Clinical applications of prenatal and postnatal therapy using stem cells retrieved from amniotic fluid. Curr Opin Obstet Gynecol 23:109–116 21386681 10.1097/GCO.0b013e32834457b1

[19] Ramachandra DL, Shaw SSW, Shangaris P, Loukogeorgakis S, Guillot PV, De Coppi P and David AL (2014). In utero therapy for congenital disorders using amniotic fluid stem cells. Front Pharmacol 5:270 25566071 10.3389/fphar.2014.00270 PMC4271591

[20] Shaw SWSWS, Blundell MPMP, Pipino C, Shangaris P, Maghsoudlou P, Ramachandra DLDL, Georgiades F, Boyd M, Thrasher AJAJ, et al. (2015). Sheep CD34+ amniotic fluid cells have hematopoietic potential and engraft after autologous in utero transplantation. Stem Cells 33:122–132 25186828 10.1002/stem.1839

[21] Shaw SWS, Bollini S, Nader KA, Gastaldello A, Mehta V, Filppi E, Cananzi M, Gaspar HB, Qasim W, De Coppi P and David AL (2016). Autologous transplantation of amniotic fluid-derived mesenchymal stem cells into sheep fetuses. Cell Transplant 25:615 28836829 10.3727/096368916X691006

[22] Veiby OP, LoCastro S, Bhatnagar P and Olsen WM (1996). Inhibition of enriched stem cells in vivo and in vitro by the hemoregulatory peptide SK&F108636. Stem Cells 14:215–224 8991541 10.1002/stem.140215

[23] Wegorzewska M, Nijagal A, Wong CM, Le T, Lescano N, Tang Q and MacKenzie TC (2014). Fetal intervention increases maternal T cell awareness of the foreign conceptus and can lead to immune-mediated fetal demise. J Immunol 192:1938–1945 24415782 10.4049/jimmunol.1302403 PMC4268439

[24] Nijagal A, Flake AW and MacKenzie TC (2012). In utero hematopoietic cell transplantation for the treatment of congenital anomalies. Clin Perinatol 39:301–310 22682381 10.1016/j.clp.2012.04.004

[25] Mold JE, Michaëlsson J, Burt TD, Muench MO, Beckerman KP, Busch MP, Lee T, Nixon DF, McCune JM, Karen P and Joseph M (2008). Maternal alloantigens promote the development of tolerogenic fetal regulatory T cells in utero. Science 322:1562–1565 19056990 10.1126/science.1164511 PMC2648820

[26] Singh KP, Gerard HC, Hudson AP, Reddy TR and Boros DL (2005). Retroviral Foxp3 gene transfer ameliorates liver granuloma pathology in Schistosoma mansoni infected mice. Immunology 114:410–417 15720442 10.1111/j.1365-2567.2004.02083.x PMC1782091

[27] Steinman RA (2002). Cell cycle regulators and hematopoiesis. Oncogene 21:3403–3413 12032778 10.1038/sj.onc.1205325

[28] Srivastava AS, Kaushal S, Mishra R, a Lane T and Carrier E (2006). Dexamethasone facilitates erythropoiesis in murine embryonic stem cells differentiating into hematopoietic cells in vitro. Biochem Biophys Res Commun 346:508–516 16764825 10.1016/j.bbrc.2006.05.130

[29] Fisher JE, Lillegard JB, McKenzie TJ, Rodysill BR, Wettstein PJ and Nyberg SL (2013). In utero transplanted human hepatocytes allows for postnatal engraftment of human hepatocytesin pigs. Liver Transpl 19:328–335 23280879 10.1002/lt.23598 PMC3600116

[30] Loukogeorgakis SP and Flake AW (2014). In utero stem cell and gene therapy: current status and future perspectives. Eur J Pediatr Surg 24:237–245 24945440 10.1055/s-0034-1382260

[31] Hayashi S, Abdulmalik O, Peranteau WH, Ashizuka S, Campagnoli C, Chen Q, Horiuchi K, Asakura T and Flake AW (2003). Mixed chimerism following in utero hematopoietic stem cell transplantation in murine models of hemoglobinopathy. Exp Hematol 31:176–184 12591283 10.1016/s0301-472x(02)01024-x

[32] Derderian SC, Togarrati PP, King C, Moradi PW, Reynaud D, Czechowicz A, Weissman IL and MacKenzie TC (2014). In utero depletion of fetal hematopoietic stem cells improves engraftment after neonatal transplantation in mice. Blood 124:973–980 24879814 10.1182/blood-2014-02-550327 PMC4126335

[33] Nijagal A, Derderian C, Le T, Jarvis E, Nguyen L, Tang Q and Mackenzie TC (2013). Direct and indirect antigen presentation lead to deletion of donor-specific T cells after in utero hematopoietic cell transplantation in mice. Blood 4595–4602 23610372 10.1182/blood-2012-10-463174 PMC3668492

[34] Bilate AM and Lafaille JJ (2012). Induced CD4+Foxp3+ regulatory T cells in immune tolerance. Annu Rev Immunol 30:733–758 22224762 10.1146/annurev-immunol-020711-075043

[35] Spence PJ and Green EA (2008). Foxp3+ regulatory T cells promiscuously accept thymic signals critical for their development. Proc Natl Acad Sci U S A 105:973–978 18198277 10.1073/pnas.0709071105 PMC2242703

[36] Burt TD (2013). Fetal regulatory T cells and peripheral immune tolerance in utero: implications for development and disease. Am J Reprod Immunol 69:346–358 23432802 10.1111/aji.12083 PMC3951896

[37] van der Net JB, Bushell A, Wood KJ and Harden PN (2016). Regulatory T cells: first steps of clinical application in solid organ transplantation. Transpl Int 29:3–11 25981203 10.1111/tri.12608

[38] Velásquez-Lopera MM, Eaton VL, Lerret NM, Correa LA, DeCresce RP, García LF and Jaramillo A (2008). Induction of transplantation tolerance by allogeneic donor-derived CD4+CD25+Foxp3+ regulatory T cells. Transpl Immunol 19:127–135 18503888 10.1016/j.trim.2008.02.003

