# Supplementary material for: Long-Term Hematopoietic Engraftment of Congenic Amniotic Fluid Stem Cells After in Utero Intraperitoneal Transplantation to Immune Competent Mice
Source: Stem Cells Dev. 2018 Apr 15;27(8):515–23. doi: 10.1089/scd.2017.0116 (PMC5910037; doi:10.1089/scd.2017.0116)
Supplement: Supplemental data [file Supp_Fig1.pdf]

Supplementary Data

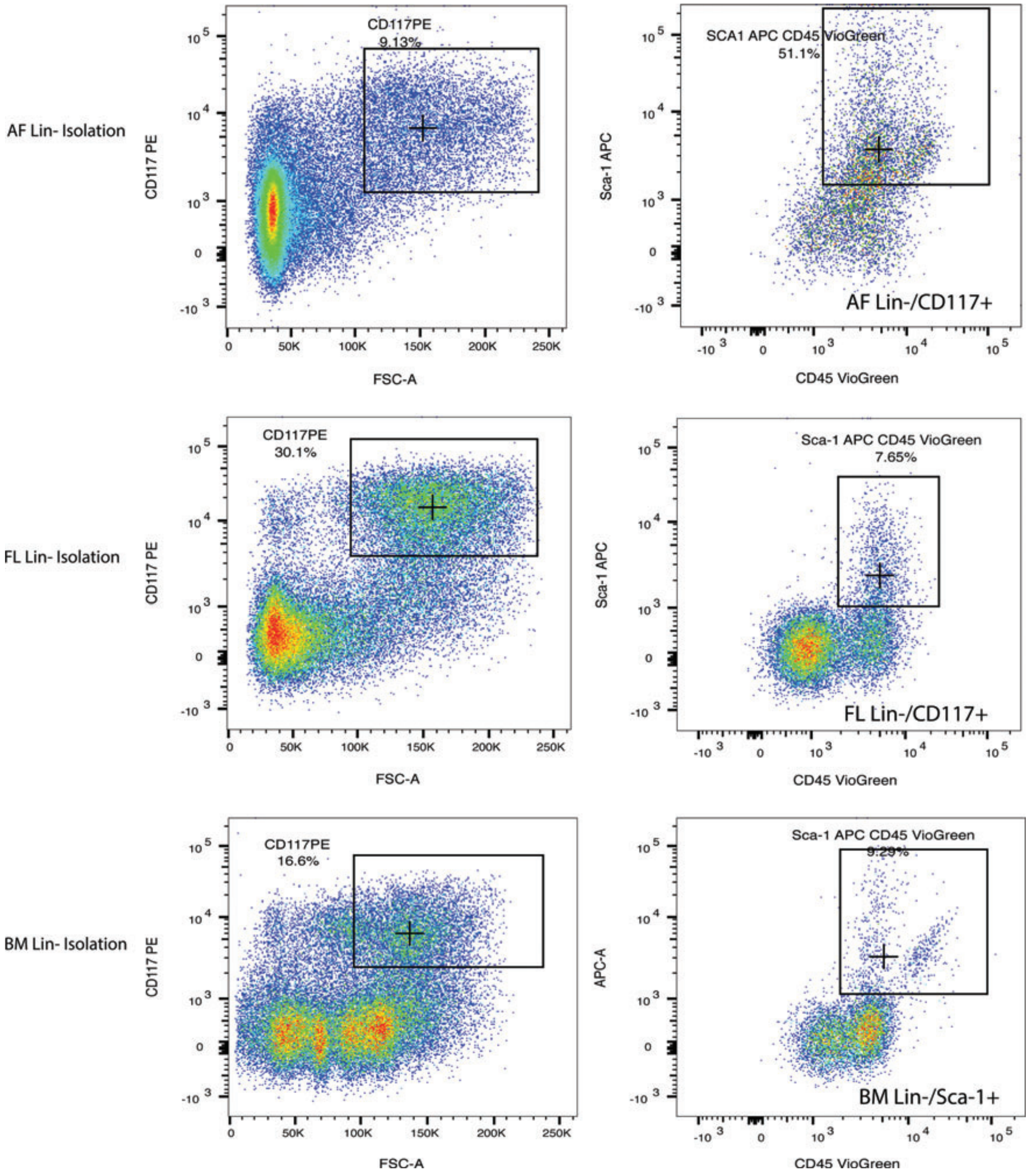

SUPPLEMENTARY FIG. S1. Flow cytometry analysis of the isolated cells from fetal liver, bone marrow and amniotic fluid showing co-expression of stem cell markers
