# Supplementary material for: Long-Term Hematopoietic Engraftment of Congenic Amniotic Fluid Stem Cells After in Utero Intraperitoneal Transplantation to Immune Competent Mice
Source: Stem Cells Dev. 2018 Apr 15;27(8):515–23. doi: 10.1089/scd.2017.0116 (PMC5910037; doi:10.1089/scd.2017.0116)
Supplement: Supplemental data [file Supp_Fig2.pdf]

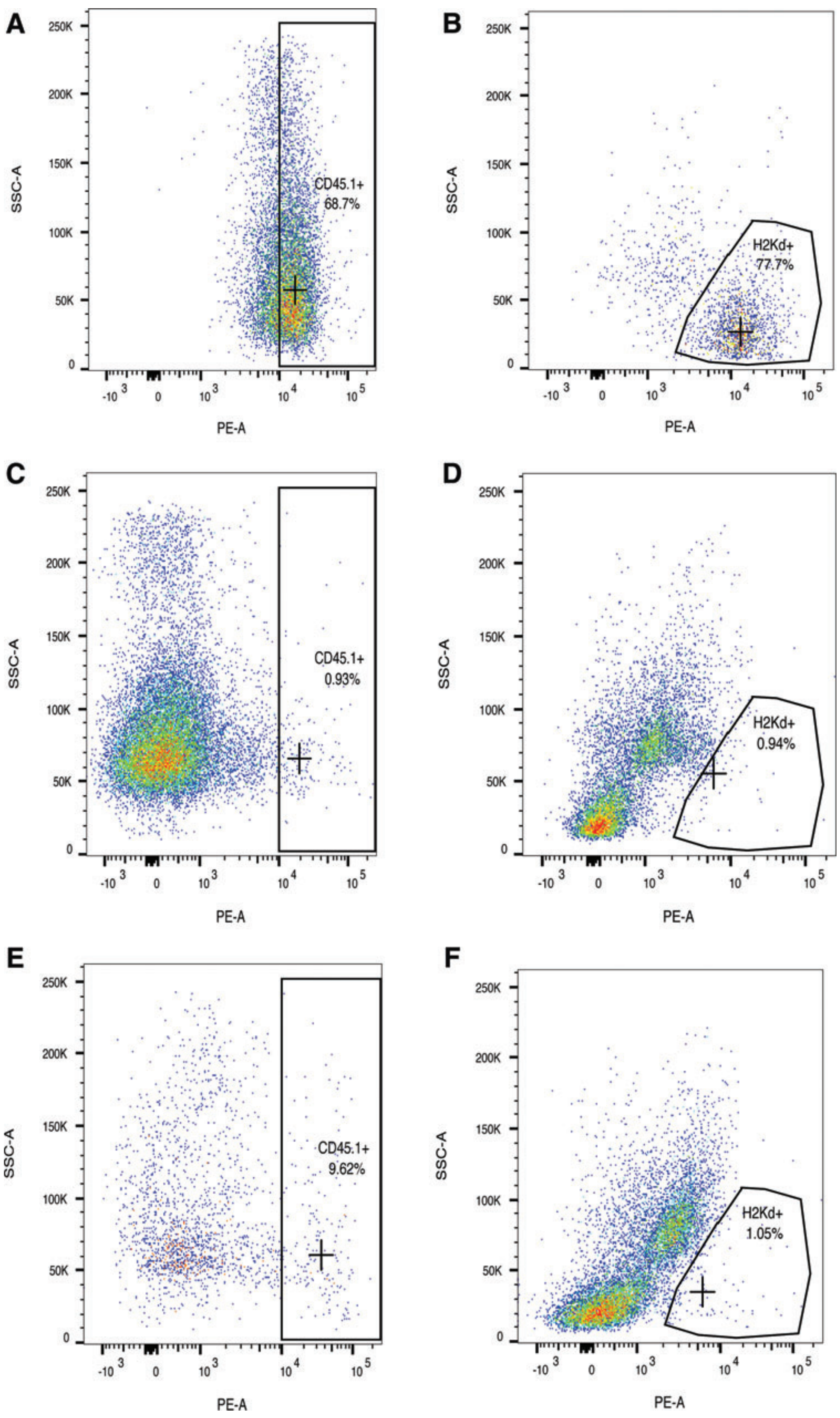

SUPPLEMENTARY FIG. S2. Flow cytometry gating strategy. **(A)** Congenic Positive Control (Blood). **(B)** Allogenic Positive Control (Blood). **(C)** Congenic Negative Control (Blood). **(D)** Allogenic Negative Control (Blood). **(E)** Congenic Representative Experimental Animal (Blood). **(F)** Allogenic Representative Experimental Animal (Blood).
