# Supplementary material for: Long-Term Hematopoietic Engraftment of Congenic Amniotic Fluid Stem Cells After in Utero Intraperitoneal Transplantation to Immune Competent Mice
Source: Stem Cells Dev. 2018 Apr 15;27(8):515–23. doi: 10.1089/scd.2017.0116 (PMC5910037; doi:10.1089/scd.2017.0116)
Supplement: Supplemental data [file Supp_Fig3.pdf]

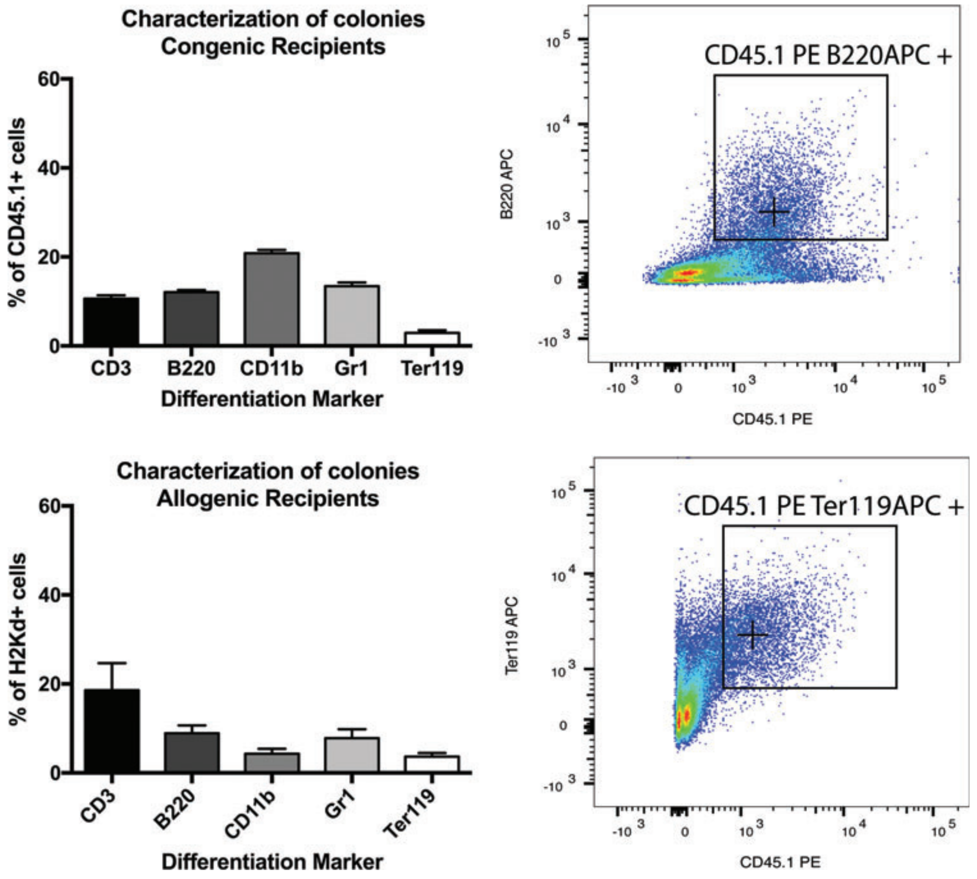

SUPPLEMENTARY FIG. S3. Flow cytometric analysis of CFUs derived from pooled colonies from congenic and allogenic recipients, showing the presence of donor markers (H2K-d, CD45.1) and expression of terminal differentiation markers. CFU, colony forming unit.
